# Evaluation of LncRNA NEAT1 and MEG3 Expression Levels in Hospitalized COVID‐19 Patients

**DOI:** 10.1155/bmri/6034615

**Published:** 2026-07-02

**Authors:** Pegah Mohammadi, Niloofar Neisi, Mojtaba Rasti, Mehdi Parsanahad

**Affiliations:** ^1^ Infectious and Tropical Diseases Research Center, Health Research Institute, Ahvaz Jundishapur University of Medical Sciences, Ahvaz, Iran, ajums.ac.ir; ^2^ Department of Medical Virology, School of Medicine, Ahvaz Jundishapur University of Medical Sciences, Ahvaz, Iran, ajums.ac.ir

**Keywords:** COVID-19, gene expression, LncRNA MEG3, LncRNA NEAT1, real-time PCR

## Abstract

**Introduction:**

The novel coronavirus known as SARS‐CoV‐2 was initially discovered in Wuhan, China, in December 2019, as the cause of COVID‐19, according to the World Health Organization in March 2020. Today, the role and function of LncRNAs in the pathogenesis of viral diseases and their diagnosis and treatment have been widely investigated. This study is aimed at evaluating the expression levels of LncRNA NEAT1 and LncRNA MEG3 in hospitalized COVID‐19 patients compared to healthy controls.

**Methods:**

This study is a case‐control. Patients′ serum samples were taken from patients with COVID‐19 hospitalized in the covid department of Razi Hospital in Ahvaz, as 30 serum samples from healthy people served as the control group and the case group. RNA extraction was performed for all samples. Then, the expression level of LncRNA NEAT1 and LncRNA MEG3 genes in COVID‐19 patients and the control group was done using real‐time PCR technique. Finally, all the obtained results were analyzed using REST software.

**Results:**

According to the obtained results, the case group′s LncRNA NEAT1 gene expression level was higher than that of the control group, was not significant (*p* = 0.217), and COVID‐19 patients′ LncRNA MEG3 gene expression was reduced in COVID‐19 patients compared to controls (*p* = 0.001).

**Conclusion:**

The expression of LncRNA MEG3 was significantly downregulated in COVID‐19 patients (*p* = 0.001), suggesting its potential role in viral immune evasion. Although LncRNA NEAT1 expression was elevated in patients, this increase was not statistically significant (*p* = 0.217). Therefore, only LncRNA MEG3 appears to be a promising biomarker for COVID‐19 progression, whereas the role of LncRNA NEAT1 requires further investigation in larger cohorts.

## 1. Introduction

In December of 2019, an unprecedented cluster of pneumonia cases with an indeterminate etiology emerged in Wuhan, Hubei Province, China. In response, Chinese authorities and medical researchers initiated swift interventions to contain the outbreak and commenced investigations into the causative agents. The pathogen was identified as a new coronavirus. The virus, the coronavirus, initially designated as the 2019 novel coronavirus on a provisional basis, the virus was named SARS‐CoV‐2 by the International Committee on Classification of Viruses. Corona virus 2019, or COVID‐19, is the name of the illness that this virus causes. [1]

Coronaviruses are enveloped viruses characterized by positive‐sense single‐stranded RNA, originating from animals, and classified within the order Nidovirales, family Coronaviridae, and subfamily Coronavirinae, and based on genetic characteristics, this subfamily is classified into four genera: alpha coronavirus, beta coronavirus, gamma coronavirus, and delta coronavirus [2]. SARS‐CoV‐2 is classified within the beta coronavirus group, yet it exhibits a strong affinity for The lower respiratory system and lungs are affected by SARS‐CoV‐2, which is recognized as a pathogen that possesses a higher transmission and infectivity rate than SARS and MERS [3, 4]. The disease typically has an incubation period ranging from 3 to 7 days. Approximately 80% of cases are either mild or asymptomatic, whereas 15% are classified as severe, and 5% progress to a critical state necessitating mechanical ventilation. The three main infection courses encompass mild illnesses characterized by upper respiratory symptoms, nonsevere pneumonia, and severe pneumonia that may lead to acute respiratory distress syndrome and multiple organ failure [5]. Although the process of the pathogenesis of the disease is not well known yet, the studies conducted so far have shown that the number of leukocytes of the lymphoid line decreases and the amount of inflammatory cytokines increases in patients infected with this virus. Probably, excessive inflammatory responses of the innate immune system to the virus, pneumonia, and damage to the lung tissue is the main cause of high mortality of this virus [6]. In patients with COVID‐19, extensive lung damage is seen, which leads to acute respiratory distress syndrome in some patients. Changes in the balance of immune cells and uncontrollable production of various cytokines are the causes of acute respiratory distress syndrome (ARDS). Therefore, what is very important in the pathogenesis of this disease is the defense reactions of the immune system against the virus [7].

A growing body of research has indicated that long noncoding RNAs (LncRNAs) participate in numerous biological regulatory processes, including inflammation, cellular functions, and immune disorders, and they are crucial in the development of various diseases, such as viral infections and disease progression. In summary, LncRNAs are becoming recognized as significant regulators of severe inflammatory responses and thrombosis in individuals with COVID‐19, thus contributing to the persistence of viral infections [8–10]. Nevertheless, the characteristics and functional mechanisms of these LncRNAs in the context of COVID‐19 remain unclear. With these interpretations, the main mechanism of pathogenesis and lung injury in individuals diagnosed with COVID‐19 is characterized by the disruption of the balance in immune responses and the creation of a cytokine storm. Considering that LncRNA NEAT1 has been reported to enhance Type I interferon (IFN) responses through the RIG‐I‐like receptor pathway [11], whereas LncRNA MEG3 modulates inflammatory cytokine expression via regulation of TLR4 signaling and the NF‐*κ*B pathway [12], there is a possibility that these LncRNAs play the role in the development of cytokine storm, pulmonary injury, and respiratory distress syndrome in these patients, and therefore, detecting their presence and expression as a biomarker will play a role in disease prognosis [11]. Therefore, this study is aimed at evaluating the expression levels of LncRNA NEAT1 and LncRNA MEG3 in hospitalized COVID‐19 patients compared to healthy controls.

## 2. Material and Methods

### 2.1. Specimen Collection

This research employs a case‐control design, 60 patients with COVID‐19 admitted to the Razi Hospital in Ahvaz between June and July 2021 were consecutively enrolled after obtaining informed consent. This study was approved by the Ethics Committee of Ahvaz Jundishapur University of Medical Sciences, with the approval code: IR.AJUMS.REC.1400.155. Written informed consent was obtained from all participants. All procedures were performed in accordance with the Declaration of Helsinki.

The definitive criterion for infection and inclusion in the study for the case samples was the positive real time PCR test on the nasopharyngeal and oropharyngeal swab samples of these patients and confirmation by an infectious disease specialist, and 30 control group samples from healthy individuals without respiratory symptoms. It was obtained from the negative result of the real time PCR test and the confirmation of the absence of the disease of COVID‐19. Exclusion criteria for both case and control groups were the history of asthma, allergies, autoimmune diseases, and any inflammatory diseases. In addition, the demographic information of both groups was obtained in the form of a designed questionnaire. The peripheral venous blood was collected from each patient and control group. Following the collection process, the blood samples underwent centrifugation at 4°C at 2000 RPM for a duration of 5 min to separate the plasma, which was subsequently preserved at −70°C until analysis.

### 2.2. RNA Extraction and Determination

The extraction was performed using 200 *μ*L of plasma samples with the High Pure Viral Nucleic Acid Extraction Kit (Roche Life Science), adhering to the manufacturer′s guidelines. The synthesis of cDNA for the RNA genome was executed in a 20 *μ*L reaction mixture that included 4 *μ*L of 5x reaction buffer, 2 *μ*L of a mixed dNTP solution, 1 *μ*L of RT‐MULV enzyme (Fermentas, Germany), 2 *μ*L of random hexamer (Fermentas), 7 *μ*L of RNA template, and 4 *μ*L of RNase/DNase‐free water. The mixture was incubated at 25°C for 10 min, at 42°C for 60 min, and finally at 70°C for 10 min. The relative expressions of LncRNA NEAT1 and LncRNA MEG3 in the plasma of both patient and healthy control groups were assessed using reverse transcription‐quantitative polymerase chain reaction (RT‐qPCR). The quantitative real‐time PCR reactions and fluorescence measurements were conducted utilizing a specific primer set: LncRNA NEAT1, forward (5 ^′^‐>3 ^′^): TGTCCCTCGGCTATGTCAGA; reverse (5 ^′^‐>3 ^′^): GAGGGGACGTGTTTCCTGAG. LncRNA MEG3, forward (5 ^′^‐>3 ^′^): TCGACAAAGACTGACACCCC; reverse (5 ^′^‐>3 ^′^): TCGACAAAGACTGACACCCC. GAPDH, forward (5 ^′^‐>3 ^′^): TGACCACAGTCCATGCCATCAC; reverse (5 ^′^‐>3 ^′^): GCCTGCTTCACCACCTTCTTGA. The amplification was carried out using the Step One Real‐Time PCR System (ABI, United States), following the cycling protocol: 95°C for 2 min (activation of DNA polymerase), succeeded by 40 cycles of 95°C for 20 s (denaturation) and 60°C for 30 s (annealing and extension). All reactions were conducted in duplicate with suitable negative controls. The threshold cycle (CT) values for each gene were recorded for quantitative analysis. GAPDH was used as the internal control gene for normalization of target gene expression. The relative expression of the genes was calculated using ΔCT and 2‐ΔCT, based on the comparison between the CT value of the target gene and that of GAPDH.

### 2.3. Statistical Analysis

Rest 2009 software, excel software and SPSS V23 were used for analysis of gene expression. Quantitative variables were calculated using mean ± SD. The Student′s *t*‐test test was used to calculate statistical differences between the means of target gene expression in treated and control samples. *p* values less than 0.05 (*p* < 0.05) were considered as significant difference.

## 3. Results

In brief, a total of 60 patients were included in the study, comprising 26 females (43.3%) and 34 males (56.6%) and 30 control samples (12 females [40%] and 18 males [60%]) were healthy control without respiratory symptoms. The mean age of COVID‐19 patients was 52.69 ± 14.1 years, which was significantly higher than that of healthy controls (44.75 ± 13.09 years; *p* = 0.012). Among the 60 hospitalized patients, 5 people (8.3%) had hypertension, 4 people (6.6%) had diabetes mellitus, and 2 people (3.3%) had hyperlipidemia. The clinical and demographic characteristics of the study participants are summarized in Table 1.

**Table 1:** Clinical and demographic characteristics of study participants.

Parameter	COVID‐19 patients (*n* = 60)	Healthy controls (*n* = 30)	*p*
Age (years), mean ± SD	52.69 ± 14.1	44.75 ± 13.09	0.012
Sex, *n* (%)			0.752
Male	34 (56.7)	18 (60.0)	
Female	26 (43.3)	12 (40.0)	
Comorbidities, *n* (%)			
Hypertension	5 (8.3)	0	—
Diabetes mellitus	4 (6.7)	0	—
Hyperlipidemia	2 (3.3)	0	—
Hospitalization status, *n* (%)			
Hospitalized	60 (100)	—	—

*Note:* *p* values were calculated using Student′s *t*‐test (age) and chi‐square test (sex). A *p* value < 0.05 was considered statistically significant.

Abbreviation: SD, standard deviation.

### 3.1. LncRNA NEAT1 Relative Expressions in Both the Patient Group and the Control Group

**Figure 1:**
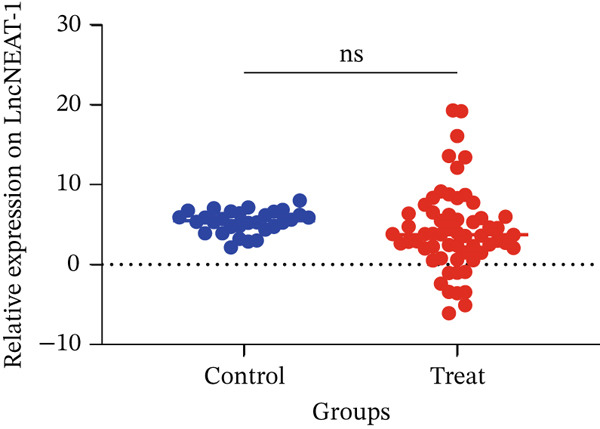
Relative expression of LncRNA NEAT1 in plasma samples of COVID‐19 patients (*n* = 60) compared with healthy controls (*n* = 30). The expression was higher in COVID‐19 patients (median: 2.176) compared with controls (median: 1), but the difference was not statistically significant (*p* = 0.217; Student′s *t*‐test). Abbreviation: ns, not significant.

The expression of LncRNA NEAT1 was found to be elevated in patients diagnosed with COVID‐19. (median value: 2.176) compared with healthy control (median value: 1), but this increase in expression was not significant (*p* = 0.217) (Figure 1).

### 3.2. LncRNA MEG3 Relative Expressions in Both the Patients and Control Group

**Figure 2:**
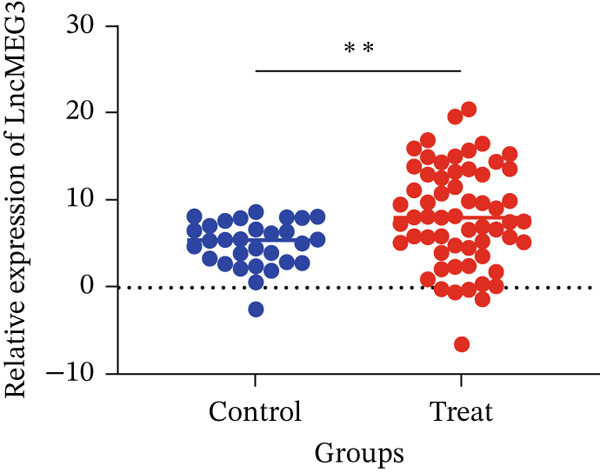
Relative expression of LncRNA MEG3 in plasma samples of COVID‐19 patients (*n* = 60) compared with healthy controls (*n* = 30). The expression was significantly lower in COVID‐19 patients (median: 0.1) compared with controls (median: 1) (*p* = 0.001; Student′s *t*‐test).  ^∗∗^p < 0.01.

The expression of LncRNA MEG3 was significantly reduced in COVID‐19 patients compared with healthy controls (*p* = 0.001) (Figure 2).

## 4. Discussion

LncRNAs are genomic elements with a length of more than 200 nucleotides, which are unable to produce proteins due to the lack of an open reading frame. By changing the structure or binding to specific proteins such as RNA‐binding proteins are crucial elements that contribute to the regulation of gene expression during the transcription phase and after that, and as a result, control various types of cell functions such as proliferation, differentiation, invasion, and apoptosis. [13].

A large number of LncRNAs have been identified as crucial elements influencing virus‐host interactions via mechanisms that are both dependent on and independent of the antiviral response. In the context of antivira response‐dependent mechanism, the regulatory functions of LncRNAs in the expression of Type I IFNs induced by the virus take place through interaction with the signaling pathway activated by pattern recognition receptors (PRRs), followed by the expression of antiviral genes downstream of the pathway. The identification of viral components by PRRs represents the initial phase in the activation of Type I IFN expression, during which various LncRNAs are involved play an important role and exert their regulatory role by affecting various components of the IFN production pathway [14].

LncRNA NEAT1 gene produces two transcripts, LncRNA NEAT1NEAT1 with a size of 3.7 kb and LncRNA NEAT1NEAT2 with a size of 23 kb. This gene participates in the regulation of gene expression, especially by keeping proteins and RNA in the nucleus [15]. In general, previous studies have shown the abnormal expression and diagnostic value of NEAT1 in different types of tumors and have confirmed the role of this LncRNA as an oncogene that causes tumor progression. The increased expression of this oncogene has been reported in different forms of cancer such as prostate, glioma, lung, breast, stomach, colorectal, liver, and kidney cancer [16]. Meanwhile, it also serves as a potential biomarker for predicting inflammation, disease severity, and respiratory distress syndrome. Also, studies show that high expression of LncRNA NEAT1 increases the risk of ARDS through induction of apoptosis and tissue destruction, especially in lung epithelial cells [17].

LncRNA MEG3 is associated with the tumor suppressor gene P53. Recent research shows that LncRNA MEG3 plays a role in lung injury regulation through the regulation of inflammation‐related signaling pathways and apoptosis‐related pathways such as Caspase 1 signaling pathway and JNK messenger pathway and STAT proteins. According to recent studies, LncRNA MEG3 is involved in increasing the expression of inflammatory cytokines CRP, IL‐1*β*, IL‐6, and MCP‐1, which in turn intensifies the inflammatory response, and is also likely to mediate cell apoptosis in major organs including the kidney and heart. Which will result in the damage of several organs [18].

The research conducted by Rodrigues et al. [19] demonstrated that the expression of the LncNEAT1 gene in saliva and nasopharyngeal swab samples from COVID‐19 patients was found to be significantly elevated when compared to the control group. By analyzing the expression of LncNEAT1 and their association with cytokines IL‐6, CCL2, and TNF‐*α* in the context of COVID‐19, Abbasi‐Kolli et al. [20] suggested that the expression level of LncNEAT1 was markedly elevated in patients with severe and moderate COVID‐19 when compared to healthy controls. Furthermore, the levels of CCL2, IL‐6, and TNF‐*α* were also increased in these patients, and a positive correlation was observed between the expression of LncNEAT1 and the levels of these cytokines. In another study, Huang et al. [21] indicated that the expression level of LncNEAT1 in the Broncho alveolar lavage (BAL) samples from patients exhibiting severe symptoms was elevated compared to that of patients with mild symptoms.

Previous research indicates that intense inflammatory responses and the cytokine storm linked to inflammation may contribute to the worsening of ARDS and potentially result in mortality. LncRNAs play a role in the inflammatory cytokine storm and the inflammatory complex, which includes IL‐6, TNF‐*α*, and NLRP3.

Regarding the expression of LncRNA MEG3 gene in the study of Tao et al. [12]—in vitro (nasopharyngeal swab samples and blood) and in vivo (cell culture) and the relationship between RSV infection and the level of LncRNA MEG3 gene expression—the results showed that in patients with RSV expression LncRNA MEG3 is downregulated in nasopharyngeal samples and virus‐infected cell cultures. In fact, LncRNA MEG3 inhibits the synthesis of proinflammatory cytokines TNF‐*α* and IL‐8 by reducing TLR4 gene expression, followed by P38 MAPK and NF‐*κ*B signaling pathways, and has a protective role in the progression of RSV infections. Consistently, a recent study by Virga et al. [22] also reported dysregulation of specific LncRNAs in COVID‐19 patients, further supporting the potential role of LncRNAs as biomarkers in SARS‐CoV‐2 infection. However, the specific LncRNAs identified vary across studies, likely due to differences in patient populations, disease severity, and sample types.

With these interpretations and according to the results of various studies on the expression of the LncRNA MEG3 gene and the significant decrease in the expression of the LncRNA MEG3 in the current research, it is possible that the reduction in the expression of this gene has a protective role for the virus in the direction of the progress of the infection and escape from the inflammatory and immunological responses induced by the system have host immunity.

In general, it can be pointed out that since the process of the pathogenesis of COVID‐19 disease is variable and this virus has the potential to involve several organs in patients and different clinical manifestations in patients according to the immunity and physiological and genetics status. Therefore, the expression of various genes in these patients has undergone many, and sometimes different, changes according to the reports of numerous studies conducted in this field. In general, according to the results of the present study and taking into account the results of various studies in this field, it seems that the changes in the expression of LncRNA NEAT1 and LncRNA MEG3 genes, by affecting the defense reactions of the immune system, play an essential role in the development of the disease. Obviously, to maintain immune homeostasis, the need to carefully control the innate antiviral response to prevent the harmful effects of overactive immune system and inflammatory autoimmune diseases seems necessary, and to achieve this goal, perhaps one of the ways is paying attention to these two LncRNAs and considering them.

### 4.1. Limitation

This study had several limitations. First, the sample size was relatively small, which may result in low statistical power. Furthermore, our sample size (*n* = 60 cases, *n* = 30 controls) is relatively modest for stable ROC estimation. Consequently, it would be beneficial to conduct further research with larger sample sizes. Second, examining the connection between the expression of these two LncRNAs and the expression of target micro RNAs of these two genes. Third, measuring the expression of genes related to these two LncRNAs and especially target genes involved in immune responses. Additionally, samples were collected in June–July 2021, before widespread COVID‐19 vaccination in Iran; thus, vaccination status was not considered as a variable in this study. Fourth, there was a significant age difference between the COVID‐19 patients and healthy controls (*p* = 0.012), with patients being older on average. Age is known to influence immune responses and COVID‐19 severity; therefore, this age mismatch represents a limitation of our study. Future studies should include age‐matched control groups to minimize this confounding facto. It is recommended to compare the expression of these LncRNAs in future studies in patients with COVID‐19 with critical, severe, and mild conditions. Finally, due to the exploratory nature of this study and the limited number of LncRNAs investigated, future studies with larger sample sizes and broader LncRNA panels (e.g., using high‐throughput sequencing) are warranted to identify additional LncRNAs that may be involved in the pathogenesis of COVID‐19.

## 5. Conclusion

In conclusion, this study demonstrates that LncRNA MEG3 is significantly downregulated in COVID‐19 patients, which may contribute to viral immune evasion and disease progression. Although LncRNA NEAT1 showed increased expression in patients, the difference was not statistically significant. These findings suggest that LncRNA MEG3, but not LncRNA NEAT1, may serve as a potential biomarker for COVID‐19. Future studies with larger sample sizes are needed to validate these results and explore the functional mechanisms of these LncRNAs in SARS‐CoV‐2 infection.

## Funding

No funding was received for this manuscript.

## Conflicts of Interest

The authors declare no conflicts of interest.

## Data Availability

The data that support the findings of this study are available on request from the corresponding author. The data are not publicly available due to privacy or ethical restrictions.
