# Correction to “Evaluation of LncRNA NEAT1 and MEG3 Expression Levels in Hospitalized COVID‐19 Patients”

**DOI:** 10.1155/bmri/9847080

**Published:** 2026-10-04

P. Mohammadi, N. Neisi, M. Rasti, and M. Parsanahad, “Evaluation of LncRNA NEAT1 and MEG3 Expression Levels in Hospitalized COVID‐19 Patients,” *BioMed Research International* 2026 (2026): 6034615, 10.1155/bmri/6034615.

In the above‐mentioned article, an Acknowledgments section was omitted in error. The Acknowledgments section is shown below:

**Acknowledgments**

The authors would like to express their gratefulness to everyone who agreed to take part in the study. This study was issued from the MSc thesis of Pegah Mohammadi and supported by the Infectious and Tropical Diseases Research Center (Grant No. OG‐0021) of the Health Research Institute at Ahvaz Jundishapur University of Medical Sciences.

We apologize for this error.

